# Supplementary material for: In Vitro Determination of the Skin Anti-Aging Potential of Four-Component Plant-Based Ingredient
Source: Molecules. 2022 Nov 21;27(22):8101. doi: 10.3390/molecules27228101 (PMC9697998; doi:10.3390/molecules27228101)
Supplement: Supplementary file 1 [file molecules-27-08101-s001.zip › molecules-2021688-supplementary.pdf]

In Vitro Determination of the Skin Anti-Aging Potential of Four-Component Plant-Based Ingredient

José Quiles [1], Maria Cabrera [2], Jonathan Jones [2], Menelaos Tsapekos [1] and Nuria Caturla [2,*]

[1] Research Projects Department, Life Length SL, Calle Miguel Angel 11, 28010 Madrid, Spain
[2] Research and Development Department, Monteloeder SL, Miguel Servet 16, 03203 Elche, Spain
* Correspondence: nuriacaturla@monteleoder.com; Tel.: +34-965-68-52-75

Determination of the Active Compounds by High-Performance Liquid Chromatography (HPLC)

EY was analysed using an HPLC instrument (Agilent 1260 Infinity II; Agilent Technologies, Inc., Palo Alto, CA, USA) coupled with a photodiode array detector and equipped with a BDS Hypersil C18 (5 µm, 250 ×4.6 mm) column (Thermo Fisher, Waltham, MA, USA). Wavelength scans from 190 nm to 500 nm. Three different methods were used:

Method 1: The mobile phase was composed of acetic acid 2.5% (v/v) aqueous solution (A) and acetonitrile (B). The following multi-step linear gradient was applied: 0 min, 5% B; 20 min, 30% B; 30 min, 90% B; 35 min, 5% B; 40 min, 5% B. The initial conditions were held for 10 min. The flow rate was 1.0 mL/min, the temperature 30 °C, and an injection volume of 20 µL. For the maker compounds, the peak area was determined at a wavelength of 330nm for the phenylpropanoids verbascoside and equinacoside and 280 nm for the citrus flavone hesperidin 520 nm. The compounds were identified by comparing the retention times and ultraviolet (UV) spectra of the peaks of the HPLC/PDA chromatograms to those of commercially available standards. Both verbascoside and equinacoside were primary standard from US Pharmacopeia (USP) and Sigma-Aldrich, respectively. Hesperidin used was the European Pharmacopoeia (EP) Reference standard. EY and phenylpropanoid standard compounds were prepared with 50% (v/v) methanol solution and hesperidin standard was prepared in DMSO.

Method 2: The mobile phase was composed of acetic acid 2% (v/v) aqueous solution (A) and acetic acid 2% (v/v) methanol solution (B). The elution conditions were as follows: 0 min, 0% B; 5 min, 1% B; 30 min, 60% B; 40 min,80% B; 50 min, 90% B; 53 min, 0% B. The flow rate was 0.75 mL/min, the column temperature 25 °C, and the injection volume 20 µL. The compounds identification and their areas quantification were determined at a wavelength of 280 nm. Punicalagin a and b were identified by comparing the retention times and ultraviolet (UV) spectra of the peaks of the HPLC/PDA chromatograms to the Punicalagin (A + B mixture) primary reference standard was obtained from Phytolab. Both EY ingredient and Punicalagin standard solutions were prepared in HPLC grade water.

Method 3: The mobile phase was composed of phosphoric acid 0.05% (v/v) aqueous solution (A) and phosphoric acid 0.05% (v/v) acetonitrile solution (B). The elution conditions were as follows: 0 min, 10% B; 15 min, 28% B; 20 min, 40% B; 25 min, 90% B; 35 min, 90% B. The flow rate was 0.8 mL/min, the column temperature 25 °C, and the injection volume 20 µL. The compounds identification and areas quantification were determined at a wavelength of 200 nm. Asiaticoside B, Madecassoside and Asiaticoside were identified by comparing the retention times and ultraviolet (UV) spectra of the peaks of the HPLC/PDA chromatograms. The content of total triterpenoid glucosides (madecassoside, asiaticoside B, asiaticoside) were calculated using asiticoside primary reference standard and applying a conversion factor of 1.017 for the sum of madecassoside and asiaticoside B.

Asiaticoside primary reference standard was obtained from USP. Both EY ingredient and the standard solutions were prepared in HPLC grade DMSO.

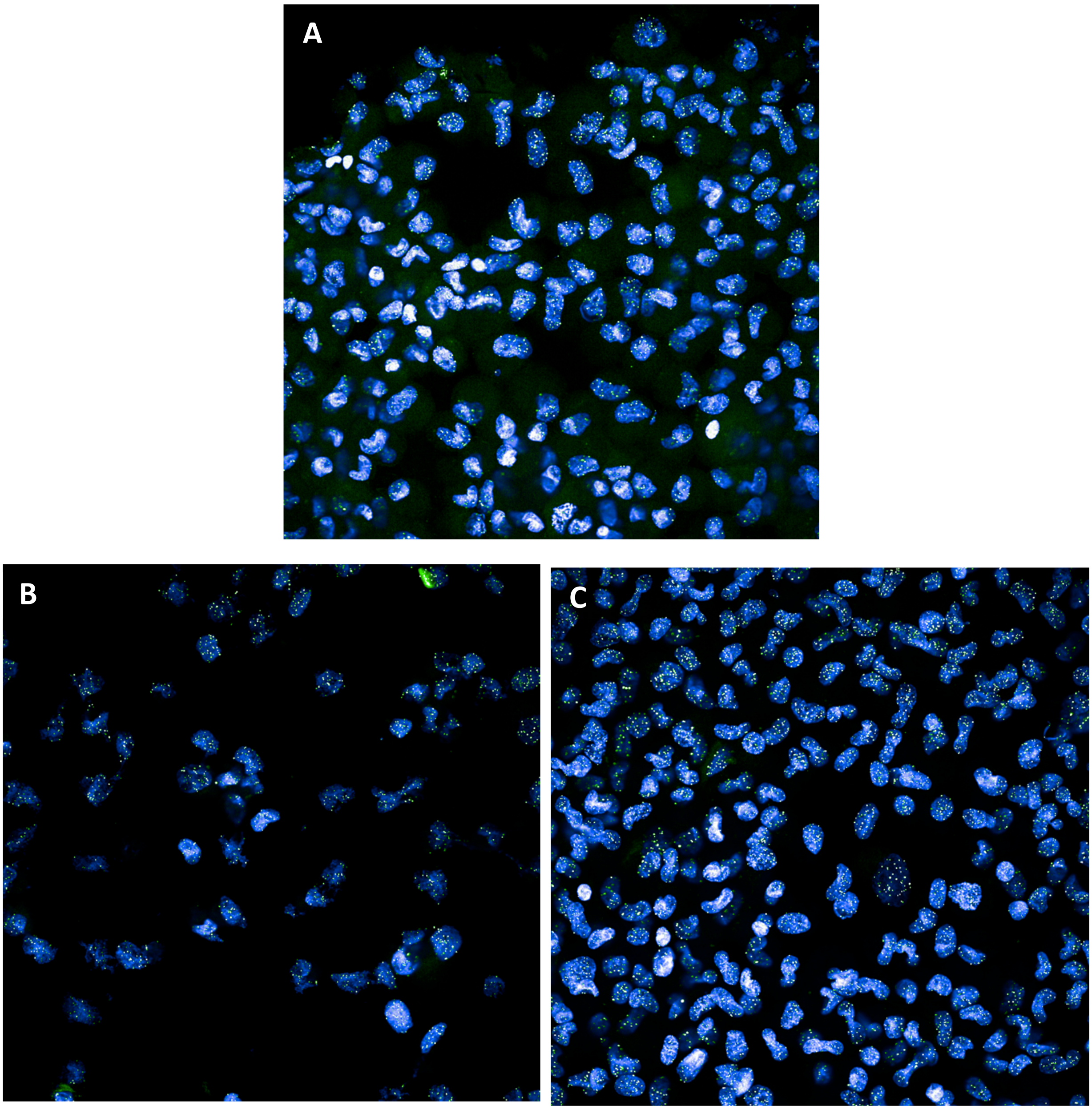

Figure S1. Representative TAT images. Both the DAPI-stained nuclei (blue) and the fluorescently labelled telomeric spots (bright spots) inside each nucleus can be seen in the images below. A) Cells at week 0; B) Cells with H_2O_2 at week 6; C) Cells with H_2O_2 at week 6 and treated with EY at 0.0001%
